# Beyond perceptual expertise: revisiting the neural substrates of expert object recognition

**DOI:** 10.3389/fnhum.2013.00885

**Published:** 2013-12-27

**Authors:** Assaf Harel, Dwight Kravitz, Chris I. Baker

**Affiliations:** Laboratory of Brain and Cognition, National Institute of Mental Health, National Institutes of HealthBethesda, MD, USA

**Keywords:** expertise, object recognition, visual perception, fMRI, review, visual cortex

## Abstract

Real-world expertise provides a valuable opportunity to understand how experience shapes human behavior and neural function. In the visual domain, the study of expert object recognition, such as in car enthusiasts or bird watchers, has produced a large, growing, and often-controversial literature. Here, we synthesize this literature, focusing primarily on results from functional brain imaging, and propose an interactive framework that incorporates the impact of high-level factors, such as attention and conceptual knowledge, in supporting expertise. This framework contrasts with the perceptual view of object expertise that has concentrated largely on stimulus-driven processing in visual cortex. One prominent version of this perceptual account has almost exclusively focused on the relation of expertise to face processing and, in terms of the neural substrates, has centered on face-selective cortical regions such as the Fusiform Face Area (FFA). We discuss the limitations of this face-centric approach as well as the more general perceptual view, and highlight that expert related activity is: (i) found throughout visual cortex, not just FFA, with a strong relationship between neural response and behavioral expertise even in the earliest stages of visual processing, (ii) found outside visual cortex in areas such as parietal and prefrontal cortices, and (iii) modulated by the attentional engagement of the observer suggesting that it is neither automatic nor driven solely by stimulus properties. These findings strongly support a framework in which object expertise emerges from extensive interactions within and between the visual system and other cognitive systems, resulting in widespread, distributed patterns of expertise-related activity across the entire cortex.

## What is expertise and why is it important to study it?

Understanding the impact of experience on human behavior and brain function is a central and longstanding issue in psychology and neuroscience. One approach to this question has been to investigate people with exceptional skill, or expertise, in various domains (e.g., chess, wine-tasting, bird watching) and determine how expert processing and the neural substrates supporting it differ from those in novices. Most broadly, expertise is defined as consistently superior performance within a specific domain relative to novices and relative to other domains (Ericsson and Lehmann, 1996). For example, top soccer players such as Cristiano Ronaldo, may excel at kicking soccer balls but not at pitching baseballs.^1^ While there are many possible domains of expertise engaging diverse facets of human cognition, including perception, attention, memory, problem solving, motor coordination and action (Ericsson et al., 2006), they all provide an opportunity to study the effect of some of the most extreme and prolonged naturally occurring forms of experience on neural function.

**Figure 1:**
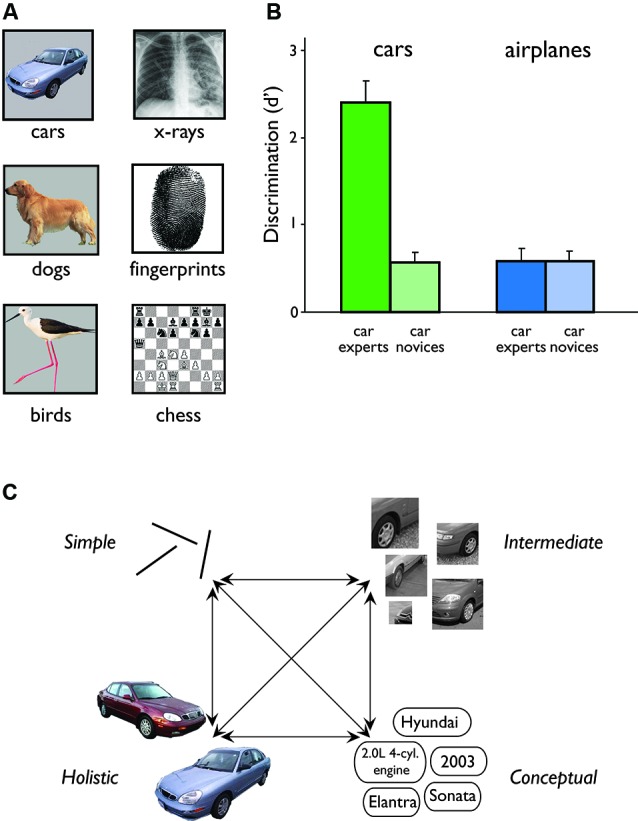
**Expert visual object recognition**. **(A)** Expertise in visual object recognition has been demonstrated in several domains, including cars (e.g., Kanwisher, 2000; Rossion and Curran, 2010; Harel and Bentin, 2013), dogs (Diamond and Carey, 1986; Robbins and Mckone, 2007), birds (e.g., Johnson and Mervis, 1997; Kanwisher, 2000), x-rays (Harley et al., 2009), fingerprints (Busey and Vanderkolk, 2005), and chess (e.g., Krawczyk et al., 2011; Bilalić et al., 2012). **(B)** Discrimination performance of car experts and car novices with cars and airplanes. Relative to naïve observers (novices), car experts are very good at telling whether two car images varying in color, view and orientation are of the same model or not. However, when these car experts have to perform a similar task with airplane images, their performance drops dramatically and is as equally poor as of novices. This exemplifies the definition of expertise as consistently superior performance within a domain relative to other people and other domains. Figure adapted from Harel et al. (2010). **(C)** A schematic representation of the different levels of visual representation that may be modified by expertise (simple features, intermediate complexity features, holistic and conceptual representations). Here we highlight the interaction between these different representational levels in the visual system. There will be further interactions between visual representations and the higher-level conceptual system representing domain-specific knowledge.

In this article, we will focus on expert visual object recognition, which is an acquired skill certain people show in discriminating between similar members of a homogenous object category, a particularly demanding perceptual task (Jolicoeur et al., 1984; Hamm and Mcmullen, 1998). Face recognition is, arguably, the quintessential example of object expertise, as almost all humans have extensive experience with faces and show remarkable face recognition abilities (Carey, 1992; Tanaka, 2001; although see evidence of significant individual differences: Bowles et al., 2009; Zhu et al., 2010; Wilmer et al., 2012). However, some individuals develop expertise for other very specific object categories. For example, ornithologists are very adept at identifying different types of birds, which all share common features (e.g., feathers, beak) but are distinct from other animals (Rosch et al., 1976; Johnson and Mervis, 1997; see Figure 1A for examples of different domains of object expertise). Such expertise may extend into even more homogenous groups such as different kinds of wading birds (Johnson and Mervis, 1997; Tanaka et al., 2005). At an even more specific level, dog show judges have enhanced recognition of individual dogs only within the particular breeds they are familiar with (Diamond and Carey, 1986; Robbins and Mckone, 2007). Similarly, car experts can distinguish between different car models (Bukach et al., 2010; Harel et al., 2010), or make ad-hoc distinctions, such as between Japanese and European cars (Harel and Bentin, 2013), even across variations in color, view and orientation. However, this car expertise does not extend to other similar domains, such as other modes of transportation (e.g., airplanes) (Figure 1B).

In this article, we primarily focus on the mechanisms that support expert visual object recognition through an examination of their neural correlates. We argue that the neural substrates of expert object recognition are not discretely localized in visual areas but distributed (e.g., Haxby et al., 2001) and highly interactive (e.g., Mahon et al., 2007), with the specific regions engaged defined by the domain of object expertise and the particular information utilized by the expert (Op de Beeck and Baker, 2010a,b; Van Der Linden et al., 2014). Through experience, this information comes to be extracted and processed through specific observer-based mechanisms both within the visual system (e.g., tuning changes) and between visual regions and extrinsic systems, key amongst which are those supporting long-term conceptual knowledge and top-down attention (Figure 1C). More broadly, we suggest that such interplay between different neural systems is a common feature of all forms of expertise. This interactive framework contrasts with the view of expertise as a predominantly sensory or perceptual skill supported by automatic stimulus-driven processes localized within category-selective visual regions in occipitotemporal cortex (e.g., Bukach et al., 2006).

We will first describe the perceptual view of visual object expertise, contrasting it with an interactive view, before focusing on the face-centric account of expert object recognition. This account has had a large influence the field of object expertise but we will highlight its major theoretical and empirical limitations. Finally, we will discuss evidence in favor of an interactive account and conclude by suggesting how this account can be generalized to explain other forms of expertise.

## The neural substrates of expert visual object recognition

### Perceptual view of expertise

What underlies expertise in object recognition? Since the hallmark of expert object recognition is making very fine discriminations between similar stimuli, one intuitive possibility is that expert object recognition primarily entails changes to sensory or perceptual processing (Palmeri et al., 2004). Thus, attaining any form of visual expertise should be supported primarily by qualitative changes in processing within specific regions of visual cortex (Palmeri and Gauthier, 2004). We refer to this notion as the perceptual view of expertise. To the extent that any changes affect the bottom-up, sensory processing of visual information, expert processing under this perceptual view is automatic and stimulus-driven, with little impact of attentional, task demands or other higher-level cognitive factors (Tarr and Gauthier, 2000; Palmeri et al., 2004).^2^

This perceptual view of expertise is supported by the experience-dependent changes in neural tuning in areas of visual cortex reported in studies of perceptual learning (e.g., Karni and Sagi, 1991), that is, “practice-induced improvement in the ability to perform specific perceptual tasks” (Ahissar and Hochstein, 2004). For example, neurons in early visual areas (V1–V4) have been reported to show stronger responses and narrower orientation tuning curves following extensive training on orientation discrimination tasks (e.g., Monkey: Schoups et al., 2001; Yang and Maunsell, 2004. Human: Schiltz et al., 1999; Schwartz et al., 2002; Furmanski et al., 2004; Yotsumoto et al., 2008; for a recent review see Lu et al., 2011). Further, long-term training with artificial objects in both human (e.g., Op de Beeck et al., 2006; Yue et al., 2006; Wong et al., 2009b; Zhang et al., 2010) and non-human primates (e.g., Kobatake et al., 1998; Op de Beeck et al., 2001; Baker et al., 2002; Woloszyn and Sheinberg, 2012) have revealed specific changes in the response of high-level visual cortex such as increases or decreases in response magnitude and increased selectivity for trained objects and task-relevant stimulus dimensions (for review, see Op de Beeck and Baker, 2010b). For example, Op de Beeck et al. (2006) trained human subjects for approximately 10 h to discriminate between exemplars in one of three novel object classes (“smoothies”, “spikies”, and “cubies”). Comparison of fMRI data before and after training revealed training-dependent increases and decreases in response across distributed areas of occipitotemporal.

### Interactive view of expertise

While these perceptual learning and training studies demonstrate changes in visual cortex with experience, such visual perceptual experience is only one aspect of real world object expertise. Objects, particularly real world natural objects embody rich information not only in terms of their appearance, but also in their function, motor affordances, and other semantic properties.^3^ Given these extended properties, the cortical representations of objects can be considered conceptual and distributed rather than sensory and localized (Mahon et al., 2007; Martin, 2009; Carlson et al., 2014). Experts and novices are distinguished by differences in these conceptual associations, since long-term real world expert object recognition is accompanied by the ability to access relevant and meaningful conceptual information that is not available to non-experts (Johnson and Mervis, 1997; Barton et al., 2009; Harel and Bentin, 2009; Gilaie-Dotan et al., 2012). However, conceptual properties of objects have not typically been manipulated in training studies such as those described above (but see Gauthier et al., 2003; Weisberg et al., 2007). Thus, in the acquisition of expertise, conceptual knowledge develops, along with other observer-based high-level factors (e.g., autobiographical memories, emotional associations) in conjunction with experience-dependent changes in perceptual processing (Johnson and Mervis, 1997; Johnson, 2001; Medin and Atran, 2004), leading to a correlation between discrimination ability and conceptual knowledge within the domain of expertise (Barton et al., 2009; Dennett et al., 2012; McGugin et al., 2012a).

A complete account of real world expert object recognition cannot ignore these factors, and must specify how stimulus-based sensory-driven processing interacts with observer-based high-level factors. For example, the expert’s increased knowledge and engagement may guide the extraction of diagnostic visual information, which in turn, may be used to expand existing conceptual knowledge. We refer to this experience-based interplay between conceptual and perceptual processing as the interactive view of expertise. This interactive view of expertise contrasts with the perceptual view of expertise (i.e., as automatic, domain-specific, and attention-invariant) and echoes a more general view of visual recognition as an interaction between stimulus information (“bottom-up”) and observer-based cognitive (“top-down”) factors such as goals, expectations, and prior knowledge (Schyns, 1998; Schyns et al., 1998; Lupyan et al., 2010). It is important to note while the interactive view does not support a strict stimulus-driven view of expert processing, it also does not suggest that the effects of experience are driven solely by top-down factors that operate independently of the perceptual processing in sensory cortex (for such a view, see Pylyshyn, 1999). Rather, we argue expertise arises from the interaction of sensory-driven and observer-based processing.

In terms of natural experience, faces perhaps best exemplify the combination of visual and conceptual properties that underlie object expertise. Faces are not only a distinct category of stimulus within which we make fine-grained discriminations, but are also typically associated with rich social, biographic and semantic information. Thus, faces seem the ideal domain to study real-world expert object recognition. And indeed, such considerations have led to an approach of studying expertise through the prism of face recognition. However, somewhat unfortunately, this approach has been dominated by the perceptual approach to expertise, focusing almost entirely on the visual aspects of processing while ignoring the influences of higher-level cognitive factors on the visual processing. We discuss this perceptually dominated face account of expertise in the following section, before presenting our interactive view of expertise in greater detail.

### The face account of expert object recognition and Fusiform Face Area (FFA)

Face perception shows a number of specific behavioral markers (e.g., stronger effects of inversion (Yin, 1969)) not typically observed for other categories of visual stimuli that are thought to reflect specialized processing mechanisms. However, it has been claimed that some of these same markers can be observed for expert object recognition, leading to the suggestion that the face processing and expertise shared a common mechanism. In their seminal paper, Diamond and Carey (1986) reported that dog experts display a similar decrement in recognition of inverted compared to upright dogs (but see Robbins and Mckone, 2007 for a failure to replicate). They reasoned that the inversion effect emerges if three conditions are met: (1) members of an object category must share a prototypical configuration of parts; (2) it must be possible to individuate the members of the category on the basis of second-order relational features (spatial relation of the parts relative to their prototypical arrangement); and (3) the observers must have the expertise to exploit such features. According to this perceptual theory of expertise, acquiring expertise in object recognition leads to a unique mode of perceptual processing, namely, transitioning from a feature-based mode of processing into what is often referred to as a “holistic” mode of processing.^4^ Consequently, this processing strategy was suggested to underlie expertise with objects in general (Gauthier et al., 2003).

In this context, many studies have compared expert and face processing to provide insight into the mechanisms of object expertise. When experts view objects from their domain of expertise, some studies have reported effects analogous to those found with faces. These include behavioral (Gauthier and Tarr, 1997, 2002), electrophysiological (Tanaka and Curran, 2001; Rossion et al., 2002; Gauthier et al., 2003; Scott et al., 2008) and neuroimaging (Gauthier et al., 1999, 2000) measures. However, other studies find conflicting results (Carmel and Bentin, 2002; Xu et al., 2005; Robbins and Mckone, 2007; Harel and Bentin, 2013) and much of evidence supporting the face account of expertise is controversial. In particular, it has been argued that the data presented in these studies is not sufficient to conclude that object expertise engages the same mechanisms as face perception (for detailed discussion see McKone and Kanwisher, 2005; McKone et al., 2007; McKone and Robbins, 2011). Here, we will focus on the neuroimaging evidence on expertise, which has predominantly investigated the role of the Fusiform Face Area (FFA; Kanwisher et al., 1997), a region in ventral temporal cortex that responds more when people view faces compared to other objects.

Broadly, there are two possible accounts of the face selectivity in FFA: (i) Stimulus driven—this region is specialized for processing faces only (Kanwisher, 2010)^5^ or (ii) Process-driven—this region is specialized for a specific computation (i.e., holistic processing) that is recruited when processing faces but can also be recruited for any object of expertise (Tarr and Gauthier, 2000). Under this process-driven account, any category of objects that share a prototypical configuration of features and require experience to discriminate between its members will engage the FFA (Gauthier and Tarr, 2002; McGugin et al., 2012b).

Supporting the process-driven account, Gauthier and colleagues reported that FFA showed a higher response to objects of expertise than to other everyday objects both in real-world experts (bird and car experts) (Gauthier et al., 2000; see also Xu, 2005) and in laboratory-trained experts with novel objects—“Greebles” (Gauthier et al., 1999). They suggested that FFA is recruited whenever expert fine discriminations among homogeneous stimuli are required. Thus, the expertise-enhanced response of FFA was suggested to be: (i) specific to categories with exemplars sharing a prototypical configuration of parts and (ii) independent of visual shape, as the increase in response was found for diverse objects of expertise (Greebles, cars, and birds). Later studies reported similar response enhancement in FFA (or in its vicinity) using chess configurations in chess experts (Bilalić et al., 2011; Righi et al., 2010). Response enhancement in FFA was also observed in children who were experts with Pokémon cartoon characters but not for Digimon characters with which they had no expertise (James and James, 2013).

However, the claim that the FFA supports expert object recognition is highly debated and is subject to much controversy. In particular, many studies have failed to find an increased response to objects of expertise in FFA: with real world expertise (Grill-Spector et al., 2004; Rhodes et al., 2004; Krawczyk et al., 2011), with short-term laboratory training (Op de Beeck et al., 2006; Yue et al., 2006) and even with the Greeble stimuli used in the original studies (Brants et al., 2011). Further, the presence of any expertise effect in FFA may reflect the perceived nature of the stimuli, particularly their resemblance to faces (Op de Beeck et al., 2006; for a discussion, see Sheinberg and Tarr, 2010).

Beyond these empirical concerns, it is important to note, that while this perceptual face-centric approach has generated a considerable body of research, it has major theoretical drawbacks for understanding the general nature of expert object recognition. These limitations are particularly evident in neuroimaging, where the theoretical discussion of the neural substrates of expert object recognition has seemingly reduced to the question of whether FFA is critically engaged in expertise (Xu, 2005; Bilalić et al., 2011; McGugin et al., 2012b) or not (Grill-Spector et al., 2004; Rhodes et al., 2004; Krawczyk et al., 2011), largely ignoring any neural signatures of expert object recognition beyond FFA that are nonetheless unique to expertise. In fact, even faces themselves elicit selective activation in many more regions than just the FFA, recruiting a whole network of cortical regions including the Occipital Face Area (OFA), Superior Temporal Sulcus (STS), Anterior Temporal Lobe (ATL), Ventrolateral Prefrontal Cortex (VLPFC), and the amygdala (for a review see Haxby and Gobbini, 2011). Further, information about faces is not restricted to these face-selective regions but is distributed across the ventral occipitotemporal cortex (Haxby et al., 2001; Susilo et al., 2010). All these regions may be highly relevant to different aspects of face expertise, for example distinguishing facial expressions supported by STS (Said et al., 2010; Pitcher et al., 2011), accessing information about unique identity invariant to visual transformations supported by ATL (Quiroga et al., 2005; Simmons et al., 2010), and processing of specialized facial features, such as the eyes, supported by VLPFC (Chan and Downing, 2011; for a review see Chan, 2013).

Thus, there is little theoretical justification for focusing solely on the FFA when many other regions, including those outside visual cortex, show the ability to support expertise with faces. Indeed, while faces are certainly a central domain of human visual expertise there are actually no a-priori reasons why the unique characteristics associated with their perceptual processing (such as holistic processing or activation of the FFA) should serve as a benchmark for all domains of object expertise. More generally, as we discuss in the next section, there is ample evidence that the neural manifestations of object expertise can be found not only in visual cortex, but also in many other cortical areas.

### Beyond Fusiform Face Area (FFA): Evidence for the broadly distributed nature of expertise

Despite the strong focus on FFA in the perceptual account of expertise, it’s clear that expertise-related activations for non-face objects are found outside FFA and even outside other face-selective regions. In fact, even the early fMRI studies of Gauthier and colleagues revealed expertise-related activations in the face-selective OFA and in other regions of occipitotemporal cortex including object-selective Lateral Occipital Complex (LOC; Malach et al., 1995), and scene-selective Parahippocampal Place Area (PPA; Epstein and Kanwisher, 1998). Subsequent fMRI studies of expert object recognition also reported expertise-specific activity outside of FFA (Harley et al., 2009; Krawczyk et al., 2011), and long-term training with artificial objects has been reported to elicit changes in many parts of occipitotemporal cortex (Op de Beeck et al., 2006; Yue et al., 2006; Wong et al., 2009b; Brants et al., 2011; Wong et al., 2012) as well as in areas outside visual cortex such as STS (Van Der Linden et al., 2010), posterior parietal cortex (Moore et al., 2006) and prefrontal cortex (Moore et al., 2006; Jiang et al., 2007; Van Der Linden et al., 2014).

**Figure 2:**
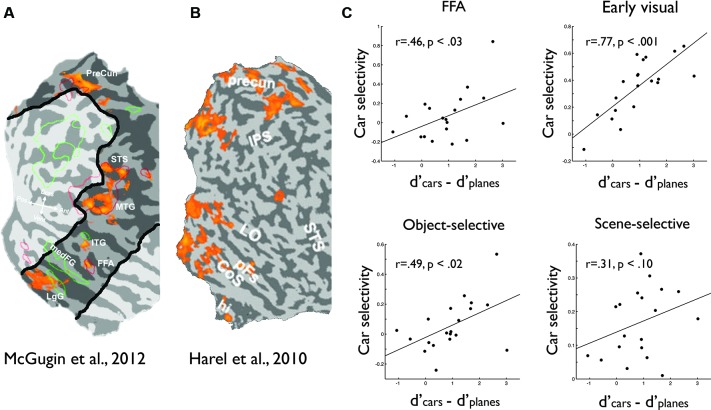
**Widespread distributed effects of expertise across the cortex**. Comparison of car expertise-related effects reported by **(A)** McGugin et al. (2012b) and **(B)** Harel et al. (2010). Common regions outside FFA visible in both maps include lingual gyrus/collateral sulcus (CoS), precuneus, and STS. Importantly, the field of view used by McGugin et al. (outlined in black) did not include early visual cortex, but expertise effects were observed by Harel et al. (2010) in these areas. **(C)** Re-analyzed data from Harel et al. (2010); Experiment 1 showing correlations between behavioral car expertise (car discrimination relative to airplane discrimination) and car-selective activity (expressed as the difference between percent signal change for cars and the mean of percent signal change of the other two categories tested) in the four independently defined regions used in that study (for details see Harel et al., 2010): FFA, early visual cortex, object-selective cortex and scene-selective cortex. Together, the distributed expertise effects (a, b) and the widespread correlations between expertise and car selectivity (c) strongly suggest that the expertise effect reported by McGugin et al. reflects attentional engagement.

To test the full extent of the neural substrates of expert object recognition across the entire brain, Harel and colleagues presented car expert and novice participants with images of cars, faces, and airplanes while performing a standard one-back task, requiring detection of image repeats (Harel et al., 2010, Experiment 1). Directly contrasting the car-selective activation (cars vs. airplanes) of the car experts with that of the novices revealed widespread effects of expertise, which encompassed not only occipitotemporal cortex, but also retinotopic early visual cortex as well as areas outside of visual cortex including the precuneus, intraparietal sulcus, and lateral prefrontal cortex (Figure 2A). These distributed effects of expertise suggest the involvement of non-visual factors, such as attention, memory and decision-making in expert object recognition (Harel et al., 2010; Krawczyk et al., 2011; Bilalić et al., 2012). Note that these patterns of activation represent the *interaction* between object category and group (experts/naïve observers), that is, reflecting car-selective activity that is greater in experts relative to novices. Thus the expert modulation of early visual cortex cannot be explained away by suggesting that low-level differences in the categories compared are driving the effect (McGugin et al., 2012b). Further, the lack of a difference in activation for faces between the experts and novices argues against a general motivational explanation.

The work discussed so far has focused on the activation differences between experts and novices at a group level. However, recently it has also been suggested that the critical test of the involvement of a region in object expertise is whether its response to objects of expertise correlates with the degree of expertise (Gauthier et al., 2005; Harley et al., 2009). Using this criterion, McGugin et al. (2012b), in a high-resolution fMRI study at 7T, reported that car selectivity in FFA correlates with car expertise (but see Grill-Spector et al., 2004 for a conflicting result). While these data, if taken alone, would appear to support the process-driven account of FFA, the focus on FFA may again be misleading. Importantly, significant correlations were found in many areas outside occipitotemporal cortex including lingual gyrus, and precuneus, strongly resembling the spatial distribution of expert activations of Harel et al. (2010; Figures 2A, B). Furthermore, within visual areas, significant correlations between car selectivity and expertise were found not only in face-selective voxels but also in non-selective voxels. Overall, if correlation between degree of expertise and response to objects of expertise is the critical marker for the neural substrates of expertise, these results suggest the involvement of a number of distributed regions and suggest no privileged status of face selectivity.

While the correlation findings of McGugin and colleagues suggest widespread effects of expertise, due to the nature of the high-resolution scanning the imaged volume was restricted to parts of occipitotemporal cortex. Importantly, data was not acquired from early visual cortex, a region implicated in expertise effects by Harel and colleagues. To replicate the findings of McGugin and colleagues and see if the correlation effects extend even to early visual cortex (suggesting task-based attentional modulation of visual activity: Watanabe et al., 1998), data from Harel et al. (2010) was re-analyzed computing the correlation between a behavioral measure of expertise (pooled across car experts and novices) and the response to cars in a number of functionally-defined regions in visual cortex (Harel et al., 2012). Not only was a positive correlation found in FFA, but also in scene-selective PPA and object-selective LOC. Critically, a positive correlation was also found in early visual cortex, highlighting a general tendency across cortex for car selectivity to correlate with behavioral expertise (Figure 2C). Together, these results suggest that even when considering the specific correlation between activity and level of expertise, the neural basis of visual expertise is not relegated to specific “hot spots” in high-level visual cortex such as FFA (or any other single localized region, for that matter), but is rather manifest in a widespread pattern of activity specific to the domain of expertise, which may reflect the engagement of large-scale top-down attentional networks (Downar et al., 2001; Corbetta and Shulman, 2002).

These findings of widespread expertise effects across the cortex argue strongly against the perceptual view of expertise and instead support a framework in which a wide variety of different regions and processes generate expert performance. This characterization is in keeping with the critical role that non-perceptual factors play in distinguishing experts from novices. Having discussed the evidence for the engagement of both stimulus-driven *and* high-level cortical regions, we now turn to studies demonstrating how their interaction supports expertise.

## Beyond perception: Evidence for the interactive nature of expertise

The interactive view of object expertise proposes that expert object recognition depends on both sensory stimulus-driven processing as well as more high-level cognitive factors with a critical interaction between these processes, whereby the expert’s increased knowledge and attention guides the extraction of diagnostic visual information. Indeed, we suggest that a theory of expert object recognition cannot be complete without taking both perceptual and top-down contributions into account. Evidence for this interaction comes from behavioral and neuroimaging studies from various domains of visual expertise that involve interactions among diverse high-level cognitive processes, particularly task-based attentional engagement and domain-specific conceptual knowledge. We first focus on two of the domains of expertise that have been most intensively investigated (cars, chess), followed by a brief review of other domains of expertise, focusing in particular on spatial navigation and reading.

### Example of interactions with task-based attention in car expertise

As noted above, the expertise effects found in Harel et al. (2010), Experiment 1 are so widespread, it seems most plausible that they reflect some non-specific effect, such as the increased level of top-down engagement that the experts have with their objects of expertise. For example, experts may direct more attention to their objects of expertise (Hershler and Hochstein, 2009; Golan et al., 2014), leading both to the increased activation observed inside (Kanwisher, 2000; McKone et al., 2007) and outside (Harel et al., 2010) FFA. Thus, an alternative account is that the enhanced activation observed for objects of expertise reflects a top-down attentional effect rather than the operation of an automatic stimulus-driven perceptual mechanism (Harel et al., 2010).

**Figure 3:**
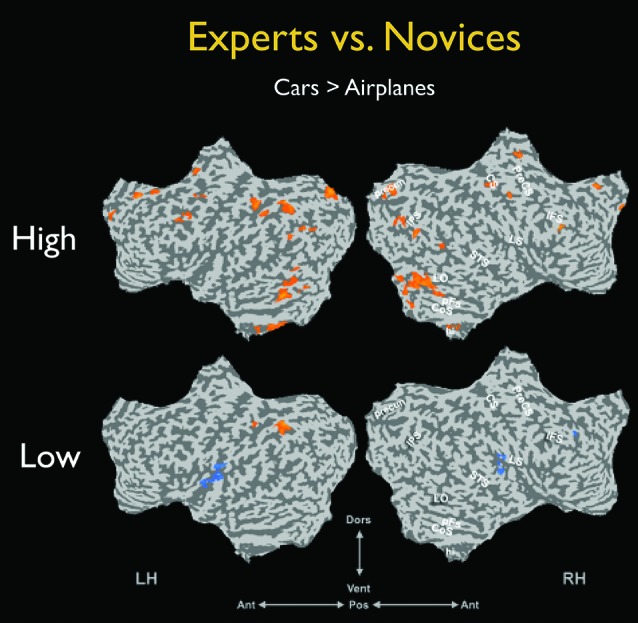
**The effect of attentional engagement on the neural correlates of expertise**. Data from Harel et al. (2010), Experiment 2 demonstrating that when experts are attending to their category of expertise (high engagement, top row), there are widespread effects of expertise compared with novices. However, these effects diminish drastically when car experts (compared with novices) engagement is drawn toward another object category (low engagement, bottom row). For further details see Harel et al. (2010).

To directly test the role of attention in expertise, Harel et al. (2010), Experiment 2 explicitly manipulated the attentional engagement of both car experts and novices. Participants were presented with interleaved images of cars and airplanes but were instructed to attend only to cars in one half of the trials, and to attend only to airplanes the other half of the trials, responding whenever they saw an immediate image repeat in the attended category only. A purely perceptual view of expertise as an automatic process would predict that the spatial extent of expert car-selective activation would be similar in both conditions, that is, irrespective of the engagement of the experts (Gauthier et al., 2000; Tarr and Gauthier, 2000). Contrary to this prediction, experts showed widespread selectivity for cars only when they were task-relevant (Figure 3, top row). When the same car images were presented, but were task-irrelevant, the car selectivity in experts diminished considerably, to the extent that there were almost no differences between the experts and novices (Figure 3, bottom row). These findings strikingly demonstrate that the neural activity characteristic of visual object expertise reflects the enhanced engagement of the experts rather than the mandatory operation of perceptual, stimulus-driven expert recognition mechanisms.

Further support for the role of attention comes from a behavioral study showing expert categorization of even car fragments involves top-down mechanisms (Harel et al., 2011). Specifically, when car experts categorized car fragments of intermediate complexity varying in their diagnostic value (Ullman et al., 2002; Harel et al., 2007), they did not utilize the information differently from novices, as might have been expected had their perceptual representations changed, but rather showed a general enhancement of response speed, indicative of a general bias or attentional effect. Further, when car experts search for cars among other common objects, they show a more efficient deployment of attention to cars relative to other object targets. The efficiency of visual search can be assessed by calculating search slopes, that is, estimating the linear increase in search speed as a function of the number of distractors displayed, with less efficient search resulting in greater increase in reaction times with increasing display size (Wolfe, 1994). Accordingly, car experts showed a shallower search slopes for objects from their domain of expertise relative to objects they are not experts with, suggesting a more efficient search (Hershler and Hochstein, 2009; Golan et al., 2014). Interestingly, the search for objects of expertise was still much less efficient than that for faces, which often result in nearly flat search slopes (Hershler and Hochstein, 2005), indicative of automatic and preattentive processing. This difference between non-face objects of expertise and faces is another demonstration that expertise in object recognition does not involve automatic perceptual processing.

While these neuroimaging and behavioral findings highlight the importance of top-down attention in expertise, experts not only direct more attention to objects of expertise, they engage in a multitude of other unique cognitive and affective processes, including accessing domain-specific knowledge. Ironically, the central role of top-down cognitive factors in object expertise can be illustrated in a domain of expertise that has been extensively studied from a perceptual perspective (e.g., Gauthier et al., 2000, 2003, 2005; Rossion et al., 2007; Bukach et al., 2010; but see Harel and Bentin, 2013). However, car experts are also more knowledgeable about cars, both about their shape and function, often possessing highly-specialized domain-specific knowledge (e.g., acceleration, horsepower). We suggest that this domain-specific conceptual knowledge interacts with and guides the extraction of visual information (Figure 1C). Several behavioral studies show that car discrimination ability is highly correlated with conceptual knowledge of cars (Barton et al., 2009; Dennett et al., 2012; McGugin et al., 2012a). These behavioral studies converge on the conclusion that car expertise integrates both visual and conceptual knowledge (for a similar conclusion, Van Gulick and Gauthier, 2013).

Finally, in addition to the fMRI studies discussed above which highlight the role of attentional engagement in car expertise, evidence for the involvement of non-visual factors can also be found in a structural MRI study. Gilaie-Dotan et al. (2012) showed that car discrimination ability is positively correlated with increasing gray matter density in prefrontal cortex. This finding is in contrast to the prediction of the perceptual view of expertise of specific changes to category-selective regions in visual cortex.

Taken together, the behavioral, structural and functional imaging studies suggest that when experts view objects from their domain of expertise, they differ from novices not only in their stimulus-driven perceptual processing of the objects, but they also direct more attention to them and access domain-specific knowledge. It is important to note that the interactive view of expert object recognition does not exclude the involvement of perceptual mechanisms in expertise that may or may not engage the FFA. Rather, changes in brain activity induced by expertise with objects reflect a multitude of interacting factors, both stimulus-driven and observer-based.

### Examples of interactions with task-based attention and prior knowledge in chess expertise

So far we have discussed evidence for the involvement of attention and conceptual knowledge in expertise, however, studies of chess expertise suggest that these two factors may operate in tandem. Chess employs multiple cognitive functions, including object recognition, conceptual knowledge, memory, and the processing of spatial configurations (Gobet and Charness, 2006). And while chess expertise has been associated with selective activations in visual cortex, and in particular FFA (Bilalić et al., 2011, but see Krawczyk et al., 2011; Righi et al., 2010), a multitude of cortical regions are reported to be active in chess experts when viewing chessboards (Bilalić et al., 2010, 2012; Krawczyk et al., 2011). Expert-related activity was found to be widespread, extending beyond visual cortex to include activations in collateral sulcus (CoS), posterior middle temporal gyrus (pMTG), occipitotemporal junction (OTJ), supplementary motor area (SMA), primary motor cortex (M1), and left anterior insula. These regions have been suggested to support pattern recognition, perception of complex relations, and action-related functional knowledge of chess objects (Bilalić et al., 2010). The exact nature of the interactions between the different areas supporting chess expertise is yet to be determined, especially how visual information is utilized and accessed by higher-level cognitive processes ubiquitous to chess, such as problem solving and decision-making.

Critically, Bilalić and colleagues demonstrated that task context and prior knowledge play an essential role in driving cortical activations in chess experts (Bilalić et al., 2010, 2011, 2012). The expert-specific pattern of activation manifested only when the task was specific to the domain of expertise (e.g., searching for particular chess pieces), and not when a comparable control task was used (i.e., a task that did not require the recognition of particular chess pieces) with identical visual input. In other words, there was little activity that distinguished experts and novices when they were not engaged, directly echoing the findings of Harel et al. (2010). Further, activity in some of the visual areas that displayed task-specific expertise effects (e.g., CoS) were also modulated by prior knowledge, demonstrated in a lower magnitude of response when the chess displays represented random, impossible chess positions relative to possible ones.

### Interactions in other domains of expertise

The interactive view of expert object recognition can be expanded to account for the neural manifestations of other types of expertise involving visual information based on the totality of the cognitive processes they recruit. In essence, the interactive view suggests that expertise is supported by a multitude of brain areas, the identity of which determined by the informational demands imposed by the particular domain of expertise. Critically, these different brain areas do not operate independently, as activity in one area is mutually constrained by activity in the others, reflecting the interactive nature of visual processing in general, and expertise in particular.

The interactive view is supported by the extensive and varied activations observed for many domains of expertise (e.g., architecture: Kirk et al., 2009; reading musical notation: Wong and Gauthier, 2010; archery: Kim et al., 2011; basketball: Abreu et al., 2012). Critically, the specific networks involved are defined by the diagnostic information for those domains. For example, professional basketball players also excel at anticipating the consequences of the actions of other players (i.e., success of free shots at a basket: Aglioti et al., 2008), reflected in activations in frontal and parietal areas traditionally involved in action observation, as well as in the extrastriate body area (EBA, a body-selective region in a occipitotemporal cortex: Downing et al., 2001), probably due to their expert reading of the observed action kinematics (Abreu et al., 2012).

Whereas many examples of visual expertise involve recognition of objects or discrete stimuli, expertise can also be found for large-scale spatial environments, for example taxi drivers navigating London (Woollett et al., 2009). Structural MRI studies have reported an increased hippocampal volume in taxi drivers relative to controls (Maguire et al., 2000; Woollett and Maguire, 2011). Importantly, these changes in hippocampus were not observed in London bus drivers with equivalent driving experience, indicating that specific navigation strategies interact with experience in producing changes to neural substrates (Maguire et al., 2006). However, in accord with the interactive view, the hippocampus is not the only region involved in navigation expertise. For example, visual inspection of landmark objects in city scenes by London taxi drivers (Spiers and Maguire, 2006) results in widespread patterns of activation along the dorsal (Kravitz et al., 2011) and ventral (Kravitz et al., 2013) visual pathways, as well as parahippocampal cortex, retrosplenial cortex, and various prefrontal structures all strongly associated with scene processing (Epstein, 2008), navigation, and spatial processing generally (Kravitz et al., 2011). Of course, all of these areas are strongly interconnected with the hippocampus, and thus constitute a network wherein multiple types of information are integrated to support complex spatial behavior.

Reading is an example of a domain in which the neural substrates supporting the interaction between conceptual and perceptual processing may be more predictable. Reading is a means of accessing the language system through vision, hence, involving the activation of multiple brain regions and interconnections supporting the processing and representation of different types of linguistic information (phonological, lexical, semantic; for a review, see Price, 2012). The visual component of reading, word processing, has been primarily linked to experience-dependent activations in ventral occipitotemporal cortex (Baker et al., 2007; Wong et al., 2009a; Dehaene et al., 2010) in a region often referred to as the Visual Word Form Area (VWFA; for a review see Dehaene and Cohen, 2011). Exemplifying the interaction between orthography and other language systems, VFWA activity following training with novel orthography was found to represent not only visual form, but also phonological and semantic information (Xue et al., 2006). In contrast to face-selective activation, which is typically stronger in the right relative to the left hemisphere, VWFA shows the opposite lateralization, with stronger responses in the left hemisphere. To explain the relative locations of face- and word-selective regions, Plaut, Behrmann and colleagues proposed a competitive interaction between face and word representation for foveally-biased cortex, constrained by the need to integrate reading with the language system that is primarily left-lateralized (Behrmann and Plaut, 2013; Dundas et al., 2013). This computational approach, which attempts to understand how higher-level, non-visual information constrains category specialization in visual areas, is likely to be a fruitful avenue for future research.

Together, these studies demonstrate that the neural substrates of visual expertise extend well beyond visual cortex, and are manifest in regions supporting attention, memory, spatial cognition, language, and action observation. Importantly, the involvement of these systems is predictable from their general functions, suggesting that expertise evolves largely within the same systems that initially process the stimuli. Overall, it is clear that more complex forms of visual expertise recruit broad and diverse arrays of cortical and subcortical regions. Visual expertise, in its broadest sense, engages multiple cognitive processes in addition to perception, and the interplay between these different cognitive systems is what unites these seemingly different domains of expertise. Notably, studying the different networks that form the neural correlates of expertise may inform us of the diverse cognitive processes involved in particular domains of expertise, as these processes are often not consciously accessible for the experts themselves (Palmeri et al., 2004).

## Summary and future directions

Real-world expertise provides a unique opportunity to study how neural representations change with experience in humans. In this article, we focused on expertise in visual object recognition, reassessing its common view as a predominantly automatic stimulus-driven perceptual skill that is supported by category-selective areas in high-level visual cortex. We propose an interactive framework for expert object recognition, which posits that expertise emerges from multiple interactions within and between the visual system and other cognitive systems, such as top-down attention and conceptual memory. These interactions are manifest in widespread distributed patterns of activity across the entire cortex, and are highly susceptible to high-level factors, such as task relevance and prior knowledge.

While the interactive framework provides a more complete account of the neural correlates of visual expertise across its diverse domains, many questions are still open. Having established the involvement of multiple cortical networks in object expertise, the next natural question is what are the relative contributions of each of these processes to the unique behavior displayed by experts. For example, examining the role of top-down attention in expertise, what is the precise effect of the high engagement of experts with their objects of expertise (inherent to real world expertise) on the perceptual processing of these objects? Using experimental paradigms that are known to affect top-down attention, such as divided attention, will allow researchers to test the extent of the involvement of top-down attention in expertise. Further, given the modulation of activation by task relevance, how do different tasks affect the neural manifestations of expertise? Similar questions can be asked about the role of conceptual knowledge in guiding perceptual processing. Of particular interest here is how accumulating knowledge over time interacts with and affects the way experts extract information from their objects of expertise.

Finally, it should be noted that the great advantage provided by studying real-world expertise—its high ecological validity—also poses a real challenge. How can the perceptual elements be teased apart from the other high-level top-down factors in real-world experts, which possess both qualities? One potential way to address this challenge is by studying long-term expertise in more controlled settings, which allow the researcher to tease apart the different factors involved in a particular domain of expertise. For example, one can study the time course of intensive, relatively short-term training with real world objects while controlling the visual input, the conceptual knowledge, and the level of engagement to manipulate the relationship between conceptual and sensory information. For example, Weisberg et al. (2007) showed that training participants to treat novel objects as tools engages action-related “tool” areas (left intraparietal sulcus and premotor cortex) that were not active before training or for objects not treated as tools. These findings demonstrate how a particular type of experience with objects is incorporated with perceptual visual information to form new object concepts. This approach can be extended to further our understanding of complex and diverse cortical networks and interactions underlying real-world expertise.

## Conflict of interest statement

The authors declare that the research was conducted in the absence of any commercial or financial relationships that could be construed as a potential conflict of interest.

## Footnotes

^1^http://www.washingtonpost.com/blogs/soccer-insider/wp/2013/08/01/soccer-and-society-cristiano-ronaldo-juggles-baseball-at-dodger-stadium/

^2^Note that the perceptual view of expertise does not claim that the task used for the training is irrelevant (in fact it is critical for inducing expertise, see Tanaka et al., 2005), but rather, that real-world experts (who are superior in within category discrimination) automatically try to individuate objects from their domain of expertise irrespective of the task at hand. In other words, once experts master the ability to individuate exemplars, they cannot view their objects of expertise without attempting to individuate them. Thus, one should distinguish between task-specific learning effects (e.g., Tanaka et al., 2005; Wong et al., 2009b) and task-dependence following expertise training (e.g., Rhodes et al., 2004).

^3^One of the most striking examples of the importance of semantic information to object recognition comes from visual associative agnosia, in which patients show intact shape processing but are unable to connect it to visual knowledge of the object (McCarthy and Warrington, 1986; Farah, 2004).

^4^Broadly defined, holistic processing refers to the calculation of the relations between the parts of the object rather than the piecemeal processing of individual object parts (for a review see Maurer et al., 2002). The term holistic is notorious in the face perception literature for its many definitions and associations (Gauthier and Tarr, 2002). In the present article, we use the term holistic in its most general, inclusive sense subsuming first- and second-order configural representations as well as holistic (integral) processing.

^5^Although the stimulus-driven account has often been linked to the notion of innate face processing, this is a separate issue. This account does not reject a role of experience, but suggests that experience contributes to the formation of stimulus-driven representations.
